# Research Frontiers in Numerical Simulation and Mechanical Modeling of Ceramic Matrix Composites: Bibliometric Analysis and Hotspot Trends from 2000 to 2025

**DOI:** 10.3390/ma19020414

**Published:** 2026-01-21

**Authors:** Shifu Wang, Changxing Zhang, Biao Xia, Meiqian Wang, Zhiyi Tang, Wei Xu

**Affiliations:** 1 Faculty of Civil Engineering and Mechanics, Kunming University of Science and Technology, Kunming 650500, China; wangshifu717@stu.kust.edu.cn (S.W.); mqwang@kust.edu.cn (M.W.); tang@kust.edu.cn (Z.T.); xuwei@kust.edu.cn (W.X.); 2 Intelligent Infrastructure Operation and Maintenance Technology Innovation Team of Yunnan Provincial, Department of Education, Kunming University of Science and Technology, Kunming 650500, China; 3 School of Intelligent Manufacturing, Changde University, Changde 415000, China; xiajinjinjin@aliyun.com

**Keywords:** ceramic matrix composites, numerical simulation, mechanical modeling, CiteSpace, bibliometrics

## Abstract

Ceramic matrix composites (CMCs) exhibit excellent high-temperature strength, oxidation resistance, and fracture toughness, making them superior to traditional metals and single-phase ceramics in extreme environments such as aerospace, nuclear energy equipment, and high-temperature protection systems. The mechanical properties of CMCs directly influence the reliability and service life of structures; thus, accurately predicting their mechanical response and service behavior has become a core issue in current research. However, the multi-phase heterogeneity of CMCs leads to highly complex stress distribution and deformation behavior in traditional mechanical property testing, resulting in significant uncertainty in the measurement of key mechanical parameters such as strength and modulus. Additionally, the high manufacturing cost and limited experimental data further constrain material design and performance evaluation based on experimental data. Therefore, the development of effective numerical simulation and mechanical modeling methods is crucial. This paper provides an overview of the research hotspots and future directions in the field of CMCs numerical simulation and mechanical modeling through bibliometric analysis using the CiteSpace software. The analysis reveals that China, the United States, and France are the leading research contributors in this field, with 422, 157, and 71 publications and 6170, 3796, and 2268 citations, respectively. At the institutional level, Nanjing University of Aeronautics and Astronautics (166 publications; 1700 citations), Northwestern Polytechnical University (72; 1282), and the Centre National de la Recherche Scientifique (CNRS) (49; 1657) lead in publication volume and/or citation influence. Current research hotspots focus on finite element modeling, continuum damage mechanics, multiscale modeling, and simulations of high-temperature service behavior. In recent years, emerging research frontiers such as interface debonding mechanism modeling, acoustic emission monitoring and damage correlation, multiphysics coupling simulations, and machine learning-driven predictive modeling reflect the shift in CMCs research, from traditional experimental mechanics and analytical methods to intelligent and predictive modeling.

## 1. Introduction

Ceramic matrix composites (CMCs) have recently become an important development direction in the field of high-temperature structural materials. Their excellent high-temperature strength, oxidation resistance, and fracture toughness allow them to demonstrate advantages in extreme environments, such as aerospace, nuclear energy equipment, and high-temperature protection systems, that traditional metals and single-phase ceramics cannot match [1,2]. As an important candidate material for hot-end components and key load-bearing structures [3], the performance of CMCs directly affects the reliability and service life of structures. Therefore, accurately predicting their mechanical response characteristics and service behavior has become a core issue in current research. However, due to their multi-phase heterogeneous characteristics, CMCs may exhibit highly complex stress distributions and deformation behavior during conventional mechanical property testing, potentially resulting in non-negligible uncertainty in the experimentally inferred key mechanical parameters (e.g., strength and modulus). In addition, the high manufacturing cost of these materials and the relative scarcity of effective experimental data further limit the precision and reliability of material design and performance evaluation based on experimental data. In this context, developing numerical simulation and mechanical modeling methods for CMCs is crucial. By constructing well-founded theoretical models, researchers can not only provide preliminary predictions and design guidance for material mechanical properties but also effectively validate and complement experimental measurements, thereby improving the rigor, accuracy, and reliability of materials research.

With the development of high-performance computing and modeling technologies, numerical simulation and mechanical modeling have become vital tools for studying the damage mechanisms and structural design of CMCs [4,5]. Methods such as continuum damage mechanics (CDM) [6], multiscale modeling [7], finite element analysis (FEA) [8], and thermo-mechanical–chemical coupling analysis [9] enable researchers to describe material stress distribution [10], crack propagation [11], and interface debonding [12,13], among other complex behaviors. These studies not only contribute to understanding the damage evolution mechanisms of materials but also provide quantitative foundations for structural optimization and lifetime prediction. Recently, data-driven modeling approaches combining machine learning, artificial intelligence [14], and high-performance computing [15,16] have emerged, providing new ideas for predicting the performance of complex composite materials. However, despite the richness of related research, the existing literature mainly focuses on specific model development and experimental validation, and a systematic quantitative analysis of the field’s knowledge structure, research hotspots, and development trends is still lacking. Recent bibliometric studies have investigated ceramic materials from different perspectives, such as functional oxide ceramics for energy-related applications. By contrast, the present study focuses specifically on ceramic matrix composites and seeks to reveal research trends related to numerical simulation, mechanical modeling, and failure mechanisms. This complementary perspective helps to clarify the development trajectory of CMCs from a mechanics-oriented viewpoint.

In this context, bibliometric and knowledge graph methods—implemented through widely used tools such as CiteSpace, VOSviewer, and Bibliometrix, and supported by analytical approaches, including co-citation analysis, co-authorship analysis, keyword co-occurrence analysis, burst detection, and thematic evolution analysis—offer effective means for investigating the evolution of research in materials science [17,18,19]. By visualizing the co-occurrence relationships of the literature, citation networks, and keyword clustering, this study aims to reveal the research themes, evolutionary paths, and frontiers of the field. This study focuses on publication trends, country and institution distribution, author collaboration networks, and keyword co-occurrence and clustering analysis, with the goal of uncovering the research patterns, knowledge structures, and evolution characteristics in the field of CMCs mechanical modeling.

Accordingly, this study systematically maps the global research landscape of numerical simulation and mechanical modeling of ceramic matrix composites from 2000 to 2025 using bibliometric and knowledge graph methods. Based on data from the Web of Science Core Collection, it analyzes publication trends, leading countries, institutions, and authors and constructs co-authorship, co-citation, and keyword co-occurrence networks to elucidate the intellectual structure and evolutionary trajectory of the field. Furthermore, this study identifies major research hotspots and emerging frontiers, providing insights that may support future methodological development and engineering applications of CMCs.

## 2. Methods and Analytical Framework Based on CiteSpace

### 2.1. Data Source

The data for this study were retrieved from the Web of Science Core Collection (SCI-Expanded). The publication period was set from 2000 to 2025, and only English-language publications were considered. A topic-based search strategy was adopted by combining material-related and method-related keywords.

The material-related keywords included “ceramic matrix composites”, “ceramic matrix composite”, “CMCs”, “ceramic-matrix composite”, “fiber-reinforced ceramics”, “oxide CMCs”, and “non-oxide CMCs”. The method-related keywords included “finite element modeling”, “numerical simulation”, “computational modeling”, “constitutive modeling”, “micromechanical modeling”, “multiscale analysis”, “damage mechanics”, “failure analysis”, “fracture mechanics”, “thermomechanical behavior”, “stress–strain response”, “mechanical behavior”, “damage evolution”, and “high-temperature performance”. These two keyword groups were combined using the Boolean operator AND in the topic (TS) field to retrieve the relevant literature. This strategy was designed to retrieve publications specifically addressing numerical simulation or mechanical modeling of CMCs.

The document types were limited to original papers and review papers. The literature search was completed on 27 October 2025. The initial search yielded 762 records. After de-duplication in CiteSpace, no duplicate records were identified, and 762 records were retained for subsequent analysis.

In CiteSpace 6.4, the time slice was set to 1 year per slice (time slice = 1). This setting enabled year-by-year network construction and visualization to identify major research themes and emerging trends in the numerical simulation and mechanical modeling of ceramic matrix composites. In CiteSpace 6.4, node types included author, institution, country, keyword, and cited reference. The selection criterion was set to g-index k=25 per time slice. No pruning was applied to sliced or merged networks (pruning sliced networks = false; pruning merged network = false).

### 2.2. Bibliometric Measurement

Using the retrieval results and citation report functions of Web of Science, we obtained the bibliometric characteristics and citation status of the retrieved documents, including publication year, country and institution of publication, citation frequency, average citation frequency, and h-index. The search results were exported in the form of “Full Record and Cited References” into CiteSpace 6.4. Visualization analysis was then performed on the authors and keywords, generating the corresponding author co-occurrence network and keyword co-occurrence clustering diagrams. This workflow enabled reproducible mapping of collaboration patterns and research themes in the retrieved literature.

## 3. Data Analysis

### 3.1. General Overview (2000–October 2025)

**Figure 1:**
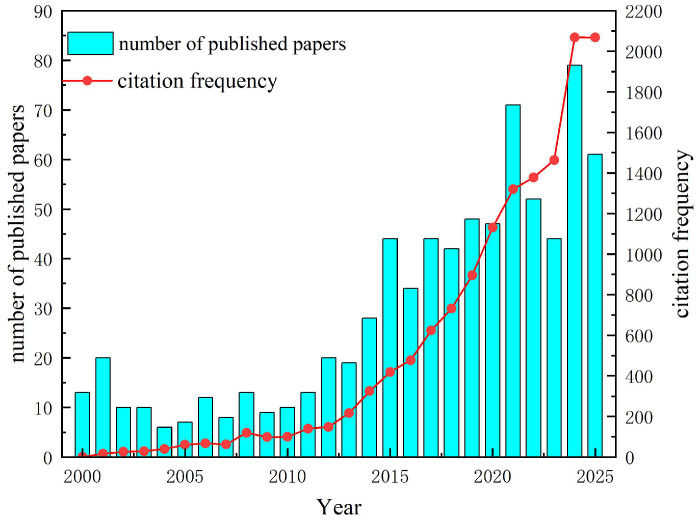
Publication volume and citation frequency of research related to the numerical simulation and mechanical modeling of ceramic matrix composites from January 2000 to October 2025. The bar chart represents the annual number of published papers, while the line graph represents the citation frequency. The left axis corresponds to publication volume, and the right axis corresponds to citation frequency.

In this study, a total of 762 documents met the inclusion criteria. According to the Web of Science citation report, these documents received 14,084 citations in total (11,657 citations excluding self-citations), with an average of 18.48 citations per item and an h-index of 54. As shown in Figure 1, publication output in this field remained relatively low from 2000 to 2013, typically ranging from single digits to slightly above ten papers per year. Since 2014, both publication output and citation activity have increased. Between 2015 and 2021, annual publications rose from approximately 30 papers in 2014 to around 70 papers in 2021, while annual citations increased in parallel. A brief fluctuation was observed in 2022–2023, during which publication output slightly decreased, whereas citation frequency continued to increase gradually. In 2024, both publication output and citation frequency rebounded, with annual publications approaching 80 and annual citations exceeding 2000, suggesting increasing academic attention and citation activity in this area. The temporal trends shown in Figure 1 reflect not only the development of numerical simulation and mechanical modeling research on ceramic matrix composites but also broader structural and contextual factors. The gradual increase in publications prior to 2020 is consistent with growing demand for high-temperature structural materials in aerospace and energy applications, as well as advances in computational mechanics and multiscale modeling techniques. The apparent fluctuations in recent years should be interpreted with caution, as bibliometric indicators are affected by publication cycles, citation time-lags, and database indexing delays. In addition, the data for 2025 include records indexed up to 27 October 2025 and, therefore, represent partial-year coverage; papers published in the most recent years have also had limited time to accumulate citations.

### 3.2. Distribution of Publications by Country

**Figure 2:**
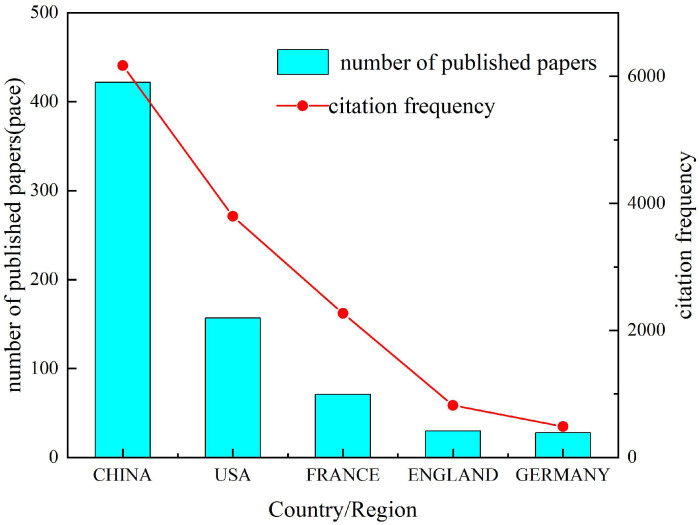
Publication volume and citation frequency of research related to the numerical simulation and mechanical modeling of ceramic matrix composites from January 2000 to October 2025 for the top 5 countries/regions. The bar chart represents the annual number of published papers, while the line graph represents the citation frequency. The left axis corresponds to publication volume, and the right axis corresponds to citation frequency.

From January 2000 to October 2025, a total of 48 countries/regions published research related to the numerical simulation and mechanical modeling of ceramic matrix composites. As shown in Figure 2 and Table 1, among the top five countries/regions in terms of publication volume, China ranks first with 422 papers, followed by the United States with 157 papers in second place. In terms of citation frequency, China’s publications have been cited a total of 6170 times, followed by the United States with 3796 citations and France with 2268 citations. France has the highest average citation frequency, at 32 citations per paper, followed by England (26.71) and the United States (24.28). Among these countries/regions, England ranks second to last in publication volume. While China leads in publication volume among these countries, its average citation frequency is the lowest, at 14.69 citations per paper.

The cross-country distribution of publications shown in Figure 2 may be associated with multiple structural factors, such as national research and innovation capacity, application-driven industrial demand (e.g., aerospace and high-temperature structural applications), and the availability of computational and experimental infrastructure. It should be noted that this study does not incorporate harmonized, field-specific cross-country investment datasets; therefore, the above factors are provided as contextual considerations rather than direct causal explanations for the observed distribution.

### 3.3. Distribution of Research Institutions (2000–October 2025)

Between 2000 and October 2025, among the top 10 research institutions publishing papers related to the numerical simulation and mechanical modeling of ceramic matrix composites, Nanjing University of Aeronautics and Astronautics ranked first with 166 papers, followed by Northwestern Polytechnical University with 72 papers in second place (see Table 2). Among the top 10 institutions, 5 are from China, 2 are from France, and 3 are from the United States. The highest citation frequency is from Nanjing University of Aeronautics and Astronautics (1700 citations), with an h-index of 22, followed closely by the Centre National de la Recherche Scientifique (1657 citations) with an h-index of 26. Notably, the highest average citation frequency is from CEA (39.39 citations), with an h-index of 17. The French National Centre for Scientific Research follows with an average citation frequency of 33.82. Nanjing University of Aeronautics and Astronautics, while ranking first in publication volume, ranks last in average citation frequency with 10.24 citations per paper.

To clarify the disciplinary backgrounds of the most productive institutions/organizations, we further summarized their Web of Science categories within the retrieved dataset. As reported in Table 3, these institutions suggest a clear interdisciplinary profile aligned with the scope of this study regarding numerical simulation and mechanical modeling of CMCs. The dominant categories are concentrated in the materials science (e.g., composites/ceramics/multidisciplinary), mechanics, and engineering-related domains (including aerospace- and defense-related organizations such as the US Department of Defense and the US Air Force). Overall, these results suggest that leading organizations in this field often integrate materials-related research with mechanics-based modeling and simulation, reflecting the cross-disciplinary nature of CMC numerical simulation and mechanical modeling research.

### 3.4. Analysis of the Main Research Content of Institutions

For each institution, “representative papers” were selected using the combined criteria of publication recency and citation impact within the institution-associated publications in our WoS dataset, so as to reflect recent developments while also capturing influential contributions. Based on these representative studies, we summarize the main research content and recent advances at the institutional level as follows. With the development of computational mechanics and high-performance computing technologies, significant progress has been made in the multiscale modeling techniques of ceramic matrix composites. Researchers have achieved multilevel modeling from the micro- to the macroscale by combining acoustic emission [20,21,22,23,24], finite element analysis [25,26], and X-ray computed tomography [27,28,29,30]. This multiscale approach not only allows for a deeper analysis of the influence of the material’s microstructure on its macroscopic mechanical behavior but also provides more accurate predictions of damage evolution and material performance [31,32]. Machine learning and artificial intelligence (AI) [33] have seen preliminary applications in the study of ceramic matrix composites, particularly in damage prediction [34], fatigue life assessment [35,36], and material performance optimization [37].

**Figure 3:**
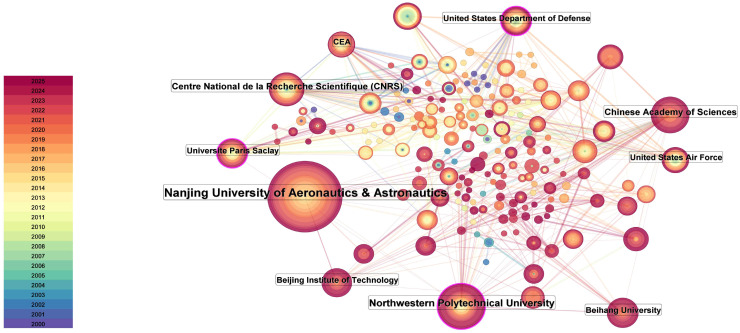
Co-occurrence of the top 10 institutions publishing research on numerical simulation and mechanical modeling of ceramic matrix composites from 2000 to October 2025. The color map in the lower-left corner represents different years with varying colors, while the node size represents the proportion of co-occurrence publications from the institutions.

As shown in Figure 3, Nanjing University of Aeronautics and Astronautics has the highest publication volume, with relatively fewer publications before 2015. The research at this institution mainly focuses on predicting the fatigue life of ceramic matrix composites (CMCs) under high-temperature conditions. Currently, the research is primarily based on an artificial intelligence (AI)-driven generative framework to predict the mechanical behavior of CMCs interface phases under complex thermo-chemical–mechanical coupling conditions. Chen et al. (2025) [38], as well as Kang et al. from Northwestern Polytechnical University, who ranked second in publication volume, have used deep learning-based image segmentation methods for damage quantification analysis [39]. Nanjing University of Aeronautics and Astronautics has conducted studies on fatigue life prediction for CMCs under high-temperature conditions, with Zhang, Sheng et al. developing a damage evolution model based on hysteresis energy dissipation, which effectively predicts the fatigue life of CMCs structures, with the maximum deviation from experimental results being less than 20% [40]. To explore the stress, strain, and damage values of CMCs under high-temperature working conditions, Xie, Haoyuan et al. simplified the periodic RVE geometric model and established a thermal–structural strength model for 2.5D woven CMCs under fine-scale temperature field influences. They discussed the effects of overall temperature levels, temperature difference forms, and the magnitude of temperature differences on the tensile curves, failure limits, damage evolution, and distribution of failure units at the point of failure for 2.5D woven CMCs [41]. In 2023, Fang, Guangwu et al. conducted research on CMCs with environmental coatings, obtaining multi-dimensional information and knowledge, including the physical properties, defects, and damage characteristics of various CMCs and environmental barrier coatings (EBCs), as well as their failure mechanisms, and established an effective failure prediction model [42]. In 2024, Chen, Mingzhu et al. further investigated the thermal stress analysis of CMCs turbine blades with environmental barriers, using finite element technology to numerically simulate and understand the failure mechanisms of CMCs, thereby improving the optimization capabilities of these components [43].

Despite these advancements, the mechanical modeling and numerical simulation of ceramic matrix composites still face several technical challenges. The fatigue behavior and thermo-mechanical coupling effects of ceramic matrix composites under high-temperature conditions remain a significant research difficulty. Existing numerical models may still face limitations in accuracy and robustness under high-temperature and complex loading conditions [44,45,46,47]. Accurately simulating the mechanical response of ceramic matrix composites under extreme conditions remains a critical technical challenge to overcome. Defects in ceramic matrix composites, such as fiber fracture [48,49], interface debonding [50,51], and micro-crack propagation [52,53], have a profound impact on material performance. Current numerical models have limitations in capturing internal defects and damage evolution. Accurately simulating the microscopic defects and macroscopic failure behavior of materials in the context of multiphysics coupling remains a challenge. Although numerical simulation techniques have been applied in the design of ceramic matrix composites, achieving the optimal synergy between material properties and structure through numerical simulations, especially balancing strength, toughness, and durability under various loading conditions, remains a major challenge in material design. The development of efficient and accurate optimization algorithms to promote intelligent material design is a key direction for future research.

Before 2020, the research at Nanjing University of Aeronautics and Astronautics mainly focused on the damage evolution of CMCs, primarily using mechanical modeling to monitor the fatigue damage evolution process. Li Longbiao and colleagues studied the stress fracture behavior of CMCs under medium-temperature random loads [54,55]. The study showed that, under random loading conditions, the stress fracture life of SiC/SiC composites shortens with increasing stress levels and random loading frequencies; it extends with increasing fiber volume fraction and shortens with increasing environmental temperature. The time required for complete interface debonding increases with fiber volume fraction, matrix crack spacing, interface debonding energy, and frictional shear stress at the slip and oxidation zones, while it shortens with increasing temperature. The team used the Budiansky–Hutchinson–Evans shear lag model (B-H-E shear lag model) to describe the microscopic stress field of damaged composites [56], monitoring the damage evolution process within the CMCs, and developed a fiber-reinforced CMCs damage evolution model based on thermomechanical fatigue hysteresis [57]. Similar to the research direction of Yang Chengpeng et al. from Northwestern Polytechnical University, this team established a simple damage-based mechanical theory to predict the nonlinear stress-strain behavior and fracture strength of orthotropic CMCs under macroscopic plane stress conditions [58].

In recent years, the research direction at Nanjing University of Aeronautics and Astronautics has remained relatively stable, closely aligned with that of Northwestern Polytechnical University. The research methods have gradually shifted from early finite element analysis (FEM), theoretical modeling, and macro-mechanical performance studies to multiscale modeling, interface damage, thermo-mechanical coupling, and intelligent monitoring. This shift marks a gradual transition from theoretical models to engineering applications. Nanjing University of Aeronautics and Astronautics utilizes artificial intelligence for fatigue life prediction of CMCs, while Northwestern Polytechnical University has adopted deep learning for damage recognition, moving toward machine learning-assisted modeling.

### 3.5. Co-Occurrence Analysis of Publications (2000–October 2025)

**Figure 4:**
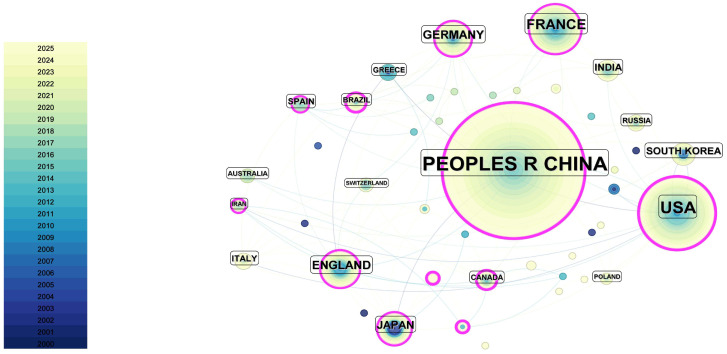
Stacked chart of publications by country on numerical simulation and mechanical modeling of ceramic matrix composites from 2000 to October 2025. The color map in the lower-left corner represents different years with varying colors, while the node size represents the publication proportion of each country.

**Figure 5:**
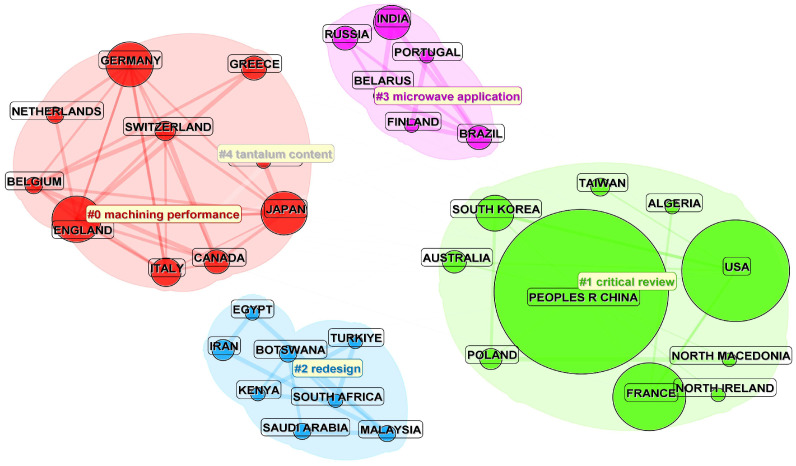
Clustering diagram of publications by country on numerical simulation and mechanical modeling of ceramic matrix composites from 2000 to October 2025. The node size represents the publication proportion, with four colors representing four research groups. Node connections indicate collaboration relationships. The # symbol preceding a number indicates the frequency of occurrence or importance of the corresponding term within the cluster.

In this study, international collaboration is inferred from co-authorship links derived from the authors’ country/region affiliations recorded in the Web of Science Core Collection. Therefore, the collaboration networks shown in Figure 4 and Figure 5 reflect cross-national co-authorship patterns within the retrieved dataset.

A total of 48 countries/regions published documents related to the numerical simulation and mechanical modeling of ceramic matrix composites. As shown in Figure 4 and Table 1, China and the United States contribute the largest shares of publications, followed by France, the United Kingdom, and Germany. Figure 5 further indicates relatively intensive international collaboration, with China and the United States occupying central positions in the collaboration network and maintaining broad links with research communities in Europe, South Korea, and Australia.

**Figure 6:**
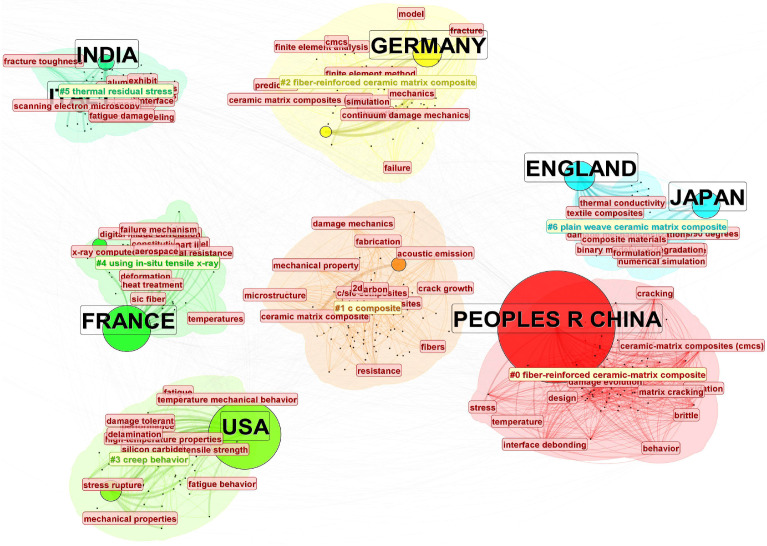
Country-based keyword overlay and clustering map of publications on numerical simulation and mechanical modeling of ceramic matrix composites (2000–October 2025). Node size indicates each country’s publication share; colors denote keyword clusters; links represent international collaboration relationships. The # symbol preceding a number indicates the frequency of occurrence or importance of the corresponding term within the cluster.

To support the interpretation of national research emphases, we additionally conducted a country-based keyword overlay and clustering analysis (Figure 6). The overlay suggests that China is more strongly associated with keyword clusters related to mechanical modeling of structural components and engineering applications, whereas the United States is more strongly associated with clusters related to multiscale simulation and mechanistic analysis. Several European countries (e.g., Germany, France, the United Kingdom, and Italy) show stronger links to keywords related to experimental studies and mechanistic characterization. In recent years, emerging research communities (e.g., Iran, Malaysia, and Turkey) have become more visible in the collaboration network, with increasing links to topics related to material modification and structural optimization. Overall, these results suggest increasing internationalization of CMCs mechanics research, while the above interpretations should be understood as associations observed in the overlay map rather than direct causal conclusions.

From Figure 5 and Figure 6, it can be observed that international collaboration in the field of ceramic matrix composites (CMCs) is relatively intensive. Both China and the United States lead in publication output and citation frequency, with strong collaborative networks extending across Europe, South Korea, Australia, and other regions. These networks are visually represented in Figure 6, where the research topics linked to these countries are clustered around themes such as mechanical modeling of structural components and engineering applications for China and multiscale simulation and mechanistic analysis for the United States.

Countries such as Germany, France, the United Kingdom, and Italy are more focused on experimental studies and mechanistic characterization of CMCs. These countries maintain stable collaborations with both China and the United States, and their academic networks are internally cohesive, as reflected in the clustering patterns seen in Figure 5 and Figure 6.

In recent years, emerging research communities from Iran, Malaysia, and Turkey have entered the field, primarily focusing on material modification and structural optimization. This shift is indicative of a rapid catch-up in research, as visualized in Figure 5, where countries like Iran and Turkey are exploring emerging areas such as material redesign and microwave applications.

Furthermore, the analysis indicates a noticeable increase in cross-regional collaborations, particularly between countries like China and Australia, the United States and France, and Europe and South Korea. This trend suggests a growing internationalization of CMCs mechanics research. Smaller countries tend to have more focused collaborations, while larger research nations often engage in broader and more diverse international partnerships. In conclusion, international collaboration is an important factor in the growth of publications related to numerical simulation and mechanical modeling of CMCs. Fostering further cooperation and academic exchange between countries is crucial for advancing the quality of scientific outputs in this field.

### 3.6. Co-Occurrence Analysis of Authors (2000–October 2025)

**Figure 7:**
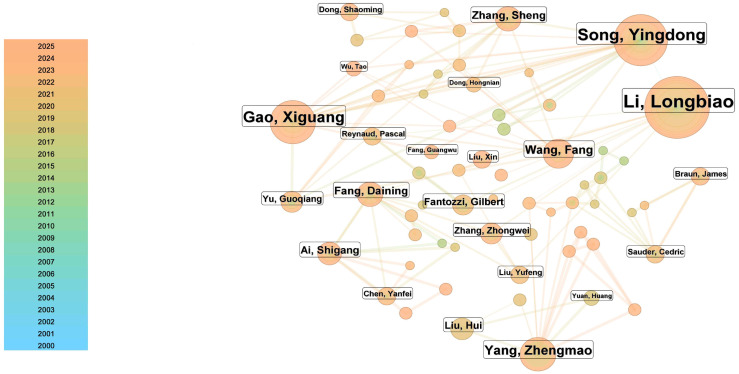
Author co-occurrence network diagram of publications on numerical simulation and mechanical modeling of ceramic matrix composites from 2000 to October 2025. The node size represents the publication proportion.

Based on the author co-occurrence network (Figure 7) and a targeted search of key authors in the Web of Science Core Collection, it was found that the team led by Li Longbiao has the highest publication output in this field, with a total of 116 articles. Their research focuses on the mechanical behavior, life prediction, and reliability design of fiber-reinforced ceramic matrix composites (CMCs) under high-temperature, high-fatigue, and damage/failure conditions. In 2022, this team proposed an innovative model based on hysteresis behavior, which can predict the crack opening and closing stresses (COS and CCS) of SiC/SiC composites under different loading and unloading conditions [59]. The results are of great practical significance for optimizing the design of CMCs and improving their performance under high temperatures and cyclic loading. In 2024, Li Longbiao further developed an innovative hysteretic cyclic constitutive model capable of accurately characterizing the influence of different matrix fragment lengths (LMFs, MMFs, and SMFs) on interface damage evolution in C/SiC composites [60]. In their 2025 study, the team applied an unsupervised clustering method (K-means algorithm) combined with acoustic emission amplitude and ring-down count parameters [61] to classify and quantitatively analyze damage types in materials, providing important experimental support for material design optimization and reliability assessment in high-temperature applications.

The team led by Song Yingdong ranks second in publication volume, with 45 articles related to numerical simulation and mechanical modeling of CMCs. This team has produced representative work in areas such as “matrix–fiber interfacial slip models,” “multilayer interfacial stress transfer,” and “fatigue life prediction models under load spectra” [62]. In 2019, they developed a new constitutive model that accounts for fiber fracture and its load-bearing capacity, proposing a more comprehensive approach to predicting the mechanical behavior of SiC/SiC composites [63]. In a 2021 study, they combined micromechanical models with mesoscale finite element models and proposed a multiscale method for predicting the fatigue life of plain-woven SiC/SiC composites [64]. Experimental validation showed that this method can effectively predict the fatigue life of the material while accounting for interfacial shear stress degradation and stochastic fiber fracture behavior, thereby providing a solid theoretical basis for material design and performance optimization. In 2025, the team proposed a constitutive model based on dynamic loading [65], which can more accurately simulate and predict the mechanical behavior of CMCs under high-rate loading conditions, demonstrating broad engineering application prospects, particularly in high-dynamic-load environments such as aerospace.

Ranking third in publication volume is Gao Xiguang, with 32 articles related to the numerical simulation and mechanical modeling of CMCs. The main research directions of this team include multiscale mechanical modeling theory and software for CMCs, as well as the high-temperature mechanical behavior of fiber-reinforced CMCs. In 2017, the team combined PFA and DIC methods to conduct an in-depth analysis of strain distribution and local failure in C/SiC composites with circular holes under tensile loading. The results showed that fiber tow arrangement and damage have a significant influence on the strain distribution and failure evolution of the material while also validating the effectiveness of the NCRCDS model [66]. In 2021, the team developed a multiscale analysis approach capable of effectively simulating the reaction–diffusion process of SiC/SiC composites under thermo-chemical–mechanical coupling conditions [67]. By introducing an equivalent diffusion coefficient model, an oxidation kinetics model, and an iterative computation scheme, the study achieved more accurate predictions of the effects of gas diffusion and oxidation reactions on composite materials.

The author collaboration network shown in Figure 7 highlights the central role of Li Longbiao and his collaborators in this field, as well as their close collaborative relationships with other researchers, particularly the strong trend of international cooperation [17,18]. Based on the network structure revealed in Figure 7, these collaborative patterns indicate an increasingly interconnected research community. In the future, as more scholars join the field and research topics continue to expand, the collaboration network is expected to become more extensive. Increasingly frequent interdisciplinary and cross-national collaborations are likely to further promote innovation and development in research on ceramic matrix composites.

### 3.7. Co-Occurrence Analysis of Keywords (2000–October 2025)

**Figure 8:**
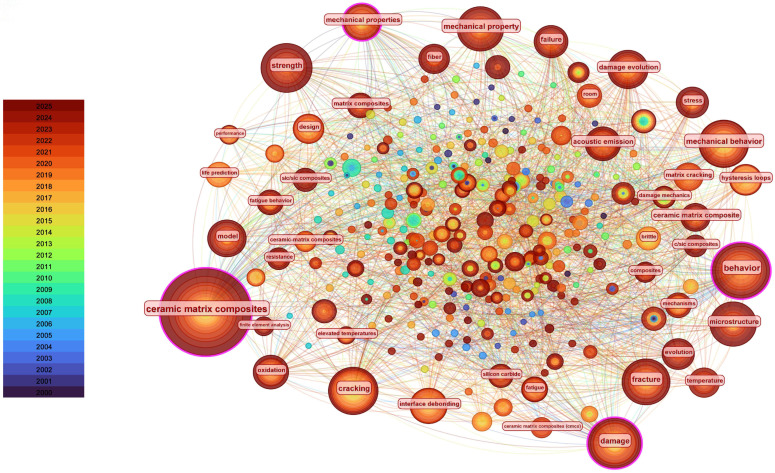
Keyword co-occurrence diagram of publications on numerical simulation and mechanical modeling of ceramic matrix composites from 2000 to October 2025. The node size represents the frequency of keyword occurrence. The larger the node, the higher the frequency of the keyword.

Using CiteSpace 6.4, a keyword co-occurrence map was generated for the research related to the numerical simulation and mechanical modeling of ceramic matrix composites (CMCs) over the past 25 years, as shown in Figure 8. The top 10 most frequent keywords are as follows: ceramic matrix composites (306), behavior (163), damage (111), mechanical property (101), strength (97), cracking (97), fracture (92), failure (69), microstructure (64), and model (58). From the keyword co-occurrence map, it is evident that the primary research hotspots related to the numerical simulation and mechanical modeling of ceramic matrix composites are focused on mechanical behavior, damage, cracking, and mechanical properties.

**Figure 9:**
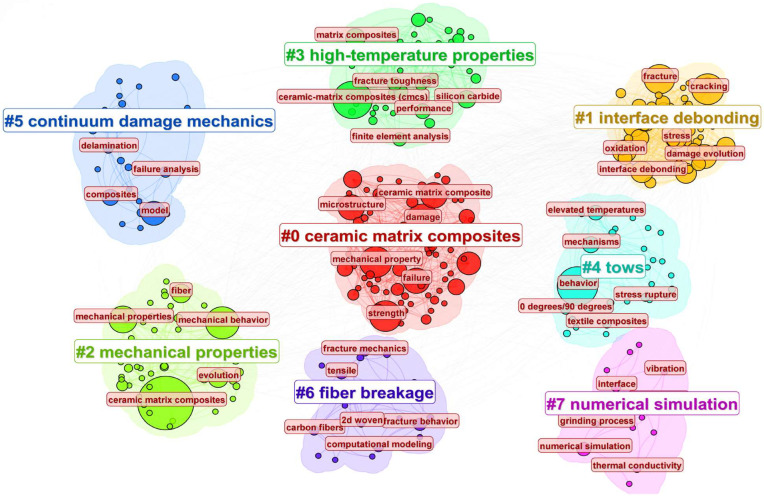
Keyword clustering diagram of publications on numerical simulation and mechanical modeling of ceramic matrix composites from 2000 to October 2025. Different colors represent different types of keyword clusters. The # symbol preceding a number indicates the frequency of occurrence or importance of the corresponding term within the cluster.

The keyword clustering map, shown in Figure 9, presents the classification of keywords into distinct thematic clusters, with good clustering quality (Modularity Q = 0.3467; Weighted Mean Silhouette = 0.7064). The ceramic matrix composites keyword focuses on the overall mechanical behavior of ceramic matrix composites (CMCs), damage and strength degradation under high temperatures [68], and the effect of microstructure on performance [69], which are the fundamental and core directions in this field. Interface debonding studies interface degradation in CMCs [70], crack propagation [71], and fatigue failure mechanisms [72], which have become important frontier topics in recent years. The mechanical properties keyword explores the macro-performance characterization of CMCs and the establishment of anisotropic models [73,74]. The high-temperature properties keyword focuses on finite element analysis and failure behavior simulations of CMCs under high-temperature service environments [75,76]. The tows keyword represents the fracture and evolution mechanisms of carbon fiber/SiC fiber bundles under stress and thermal loads [77,78]. Continuum damage mechanics refers to multiscale damage models [79,80] and fracture analysis methods [81,82], which are crucial theoretical supports for the mechanical performance prediction of CMCs. Fiber breakage refers to the impact of woven structure interface bonding on the overall mechanical properties [83,84], combined with computational modeling analysis. Numerical simulation focuses on multiphysics coupling [85], thermal conductivity prediction [86], and numerical algorithm optimization [87], representing an emerging area of development.

**Figure 10:**
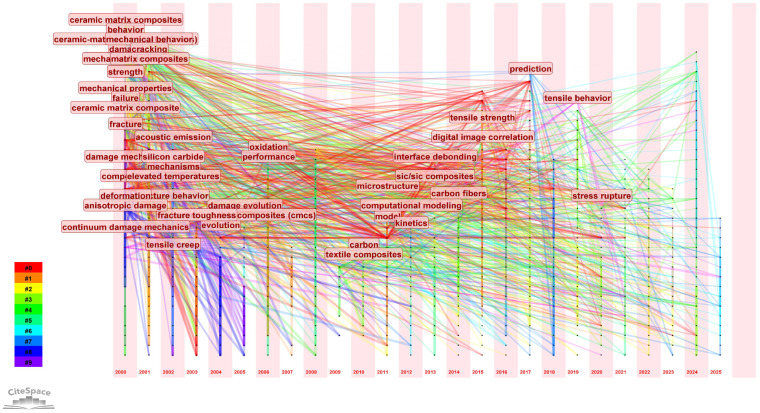
Keyword temporal evolution diagram of publications on numerical simulation and mechanical modeling of ceramic matrix composites from 2000 to October 2025. The years are labeled at the bottom of the chart, and the nodes represent keywords.

To explore the development direction of keyword research, we utilized CiteSpace’s keyword analysis function to cluster keywords and discuss their temporal evolution characteristics. As shown in Figure 10, the earliest keywords included damage, crack, strength, fiber, and mechanical properties. Subsequently, the keywords gradually evolved into model, prediction, and computational modeling. Overall, the research focus has shifted from early emphasis on macro-mechanical performance and numerical simulation fundamentals to a deeper exploration of damage and life mechanisms. The research focus has further transitioned toward intelligent monitoring and multiphysics coupling simulations [88].

## 4. Discussion

### 4.1. Overall Publication and Citation Trends

This study used bibliometric methods to analyze publications related to the numerical simulation and mechanical modeling of ceramic matrix composites (CMCs) from January 2000 to October 2025, thereby outlining the research status and evolving trends in this field. From 2000 to October 2025, the annual publication output generally increased, suggesting sustained academic attention and ongoing methodological development. Although a decline in publication output was observed in 2022–2023, this pattern should be interpreted with caution, as bibliometric indicators are influenced by multiple methodological and contextual factors rather than solely reflecting intrinsic changes in research activity. Within the analyzed window, 2024 shows the highest annual publication output and citation frequency, suggesting increasing visibility and citation activity in this area, as reflected by total citations, citations per paper (CPPs), h-index, and related indicators.

Furthermore, external factors may have influenced recent publication patterns. Global disruptions such as the COVID-19 pandemic may have temporarily altered research workflows, experimental accessibility, and collaboration modes, potentially affecting the timing of manuscript submission and publication. Fluctuations observed after 2022 may also be related to citation time-lags, database indexing delays, and partial-year coverage for 2025 (indexed up to 27 October 2025), as well as shifts in research priorities and international collaboration dynamics. However, these factors are discussed as plausible contextual influences rather than direct causal determinants, as bibliometric analysis alone cannot establish causality.

### 4.2. Country- and Institution-Level Patterns

Within the retrieved dataset (January 2000 to October 2025), China ranks first in both publication output and total citations. However, its citations per paper (CPPs) are lower than those of countries such as the United States and France. Despite China’s leading output and total citations, its CPP value ranks relatively low among the top publishing countries, indicating comparatively low per-paper citation impact. In contrast, although France ranks third in publication volume, it shows the highest CPP value among the top countries, suggesting relatively strong citation impact per item. These observations highlight the importance of considering both publication quantity and citation impact when interpreting country-level contributions.

At the institutional level, among the top 10 institutions/organizations in CMCs numerical simulation and mechanical modeling, five are from China, two from France, and three from the United States, reflecting substantial contributions from Chinese research organizations. Nanjing University of Aeronautics and Astronautics ranks first in publication output within the retrieved dataset. Representative publications from Northwestern Polytechnical University indicate an emphasis on fatigue-life prediction and damage simulation of CMCs, although its citation impact in this specific direction is comparatively less prominent. The third-ranking organization by publication volume, the Centre National de la Recherche Scientifique (CNRS), has contributed substantially to finite element analysis and mechanical modeling of CMCs microstructures.

### 4.3. Author Collaboration and Evolving Research Hotspots

In terms of authorship, the co-authorship analysis suggests the presence of approximately 200 core authors in the field of CMCs numerical simulation and mechanical modeling, based on authors appearing in the CiteSpace 6.4 co-authorship network above the selected frequency threshold. Moreover, major research teams show relatively dense collaboration links, and the two most productive teams exhibit particularly close connections in the network. These patterns suggest active knowledge exchange and an increasingly interconnected research community.

Keyword analyses for the period January 2000 to October 2025 indicate that research hotspots have evolved from early emphasis on macroscopic mechanical performance and foundational numerical simulation toward damage and life mechanisms, with more recent attention to intelligent monitoring and multiphysics-coupled simulations. This evolution reflects increasing methodological diversity and application-driven demands in CMCs mechanics research.

### 4.4. Limitations and Future Work

Several limitations should be considered when interpreting the results in Section 3. First, this bibliometric study is based on records retrieved from the Web of Science Core Collection, and the results may be influenced by database coverage and indexing policies. In addition, we intentionally included only original articles and review papers, excluding other document types (e.g., conference proceedings, meeting abstracts, editorials, and book chapters). As a result, outputs disseminated primarily through conferences or non-article formats—particularly in engineering-oriented communities—may be under-represented.

Second, citation-based indicators are subject to time-lag effects and database indexing delays. Papers published in the most recent years have had limited time to accumulate citations, and the 2025 results should be interpreted with caution because the dataset includes records indexed up to 27 October 2025 (partial-year coverage). Third, collaboration and productivity analyses rely on author names and affiliation information in bibliographic records; variations in institutional naming, multiple affiliations, and imperfect author/institution disambiguation may introduce uncertainty into network structures and institutional rankings. Fourth, bibliometric analyses are primarily descriptive and correlational; therefore, external factors are discussed as contextual considerations rather than causal determinants.

Notably, although international collaboration can be examined through co-authorship links derived from WoS affiliation information, this study does not directly quantify funding dynamics or the number of international projects due to the lack of harmonized, field-specific, and cross-country comparable project-level datasets for 2000–October 2025. Future work may integrate dedicated funding/project databases and standardized research investment indicators to explore how funding and large-scale programs relate to publication output, citation impact, and international co-authorship patterns.

Similarly, institution-level workforce statistics (e.g., researcher headcounts) are not available in a harmonized and verifiable form for cross-institution and cross-year comparisons. Although bibliometric records may provide approximate proxies (e.g., counts of unique authors affiliated with an institution), such proxies are sensitive to author name disambiguation, multiple affiliations, and inconsistencies in affiliation reporting. Therefore, we focus on publication-, citation-, and collaboration-based indicators derived from WoS records and do not interpret author-count proxies as definitive measures of institutional research capacity. Future work may integrate reliable personnel datasets or standardized institutional statistics to further assess workforce–output relationships.

## 5. Conclusions

This study used bibliometric methods to analyze the literature on the numerical simulation and mechanical modeling of ceramic matrix composites (CMCs) published between 2000 and 2025. The analysis covered aspects such as publication year, country, region, research institutions, authors, keywords, and citation frequency. The visualization results were presented using the CiteSpace 6.4 software. Over the past 25 years, the number of publications and citation frequency related to CMCs numerical simulation and mechanical modeling have shown an overall upward trend. In terms of publication volume and document quality, China, the United States, and France are the world leaders in this field. In recent years, significant progress has been made in damage identification and fatigue life prediction of CMCs under high-temperature conditions.

New mechanical models and innovative numerical simulation techniques have provided strong support for the in-depth study of CMCs mechanical properties. Using multiscale modeling methods, it is possible to precisely capture the mechanical response of three-dimensional woven CMCs under combined loading caused by off-axis bending, clearly revealing the complete damage evolution process from microscopic crack initiation to macroscopic failure. Artificial intelligence-assisted modeling and data analysis technologies enable precise prediction of the remaining life of CMCs, facilitating the development of personalized maintenance plans, reducing operation and maintenance costs, and significantly improving the reliability and service utilization of materials under extreme working conditions. Advanced numerical simulation techniques also play a critical role in the manufacturing process of complex-shaped composite components, accurately predicting the bending deformation characteristics and load-deflection response of SiC fiber three-dimensional woven preforms, providing important theoretical support for optimizing molding processes and controlling manufacturing defects.

Despite these advancements, the mechanical modeling and numerical simulation of ceramic matrix composites still face several technical challenges. First, the mechanical behavior of ceramic matrix composites under complex loading conditions requires further investigation. In particular, under non-axial tensile loading and dynamic loads, existing numerical models are still unable to fully and accurately simulate the material’s microscopic damage and macroscopic failure behavior. To address this issue, future research should focus on strengthening the integration of multiphysics coupling and multiscale modeling to improve the accuracy and applicability of these models.

Second, the simulation of microscopic defects and damage evolution under high temperatures and complex environments remains a significant challenge. Current research mainly relies on traditional numerical simulation methods, but the effects of high temperature, dynamic loading, and other factors on ceramic matrix composites are complex and variable. Future studies could explore the incorporation of machine learning and artificial intelligence techniques to promote the automation and intelligence of material modeling, particularly in damage prediction and life assessment. Furthermore, the reliability of materials and their adaptive design in high-temperature, highly corrosive environments will become a focal point of future research, especially as application demands in extreme environments such as energy sectors and aerospace continue to grow.

In conclusion, research on ceramic matrix composites has achieved breakthroughs in multiple areas, including material system innovation, manufacturing process upgrades, design method optimization, and intelligent monitoring and maintenance. Its application scenarios have gradually expanded from high-end fields such as aerospace to key industries like energy, rail transportation, and high-end equipment. In the future, with the continuous breakthroughs and deep integration of interdisciplinary technologies such as multiscale modeling, artificial intelligence, and numerical simulation, ceramic matrix composites will steadily develop toward higher efficiency; greater reliability; superior environmental adaptability; and greener, low-carbon directions, providing solid material guarantees and technical support for the transformation and upgrading of China’s high-end manufacturing industry, the independent control of major equipment, and the sustainable development of the new energy industry.

## Figures and Tables

**Table 1:** Top five countries/regions in terms of publication output regarding numerical simulation and mechanical modeling of ceramic matrix composites (2000–October 2025).

Ranking	Country/Region	Number of Published Papers	Total Citation Frequency	Citations per Paper (CPP)	h-Index
1	CHINA	422	6170	14.69	38
2	USA	157	3796	24.28	34
3	FRANCE	71	2268	32	31
4	ENGLAND	30	821	26.71	18
5	GERMANY	28	488	17.57	13

**Table 2:** Top 10 institutions by publication volume regarding numerical simulation and mechanical modeling of ceramic matrix composites from 2000 to October 2025.

Ranking	Institution	Number of Published Papers	Total Citation Frequency	Citation Frequency of Each Article	h-Index
1	Nanjing University of Aeronautics and Astronautics	166	1700	10.24	22
2	Northwestern Polytechnical University	72	1282	17.81	22
3	Centre National de la Recherche Scientifique	49	1657	33.82	26
4	Chinese Academy of Sciences	47	875	18.62	17
5	Université Paris-Saclay	35	1176	33.6	18
6	Beihang University	33	493	14.94	15
7	United States Department of Defense	33	733	22.21	16
8	CEA	31	1221	39.39	17
9	United States Air Force	30	705	23.5	15
10	Beijing Institute of Technology	29	427	14.72	12

**Table 3:** Top Web of Science categories of the top 10 institutions (based on the retrieved dataset).

Ranking	Institution	Top WoS Categories (Top 3–4)	Interpretation (Broad Areas)
1	Nanjing University of Aeronautics and Astronautics	Materials Science Composites (55); Materials Science Ceramics (46); Materials Science Multidisciplinary (40); Mechanics (27)	Materials (Composites/Ceramics) + Mechanics + Engineering/Aerospace
2	Northwestern Polytechnical University	Materials Science Multidisciplinary (25); Materials Science Ceramics (24); Materials Science Composites (19)	Materials (Ceramics/Composites) + Engineering/Materials Engineering (Interdisciplinary)
3	Centre National de la Recherche Scientifique	Materials Science Composites (14); Multidisciplinary (14); Mechanics (14)	Materials (Composites) + Mechanics + Interdisciplinary Materials/Mechanics
4	Chinese Academy of Sciences	Materials Science Ceramics (19); Materials Science Multidisciplinary (12); Materials Science Composites (10)	Materials (Ceramics/Composites) + Interdisciplinary Materials Science
5	Université Paris-Saclay	Mechanics (12); Materials Science Composites (10); Materials Science Multidisciplinary (10)	Mechanics + Materials (Composites) + Interdisciplinary Mechanics–Materials
6	Beihang University	Materials Science Ceramics (13); Mechanics (10); Materials Science Composites (9)	Materials (Ceramics/Composites) + Mechanics + Engineering/Aerospace-Oriented Research
7	United States Department of Defense	Materials Science Composites (13); Materials Science Ceramics (10); Materials Science Multidisciplinary (7)	Materials (Composites/Ceramics) + Defense-oriented Engineering R&D (Interdisciplinary)
8	CEA	Materials Science Ceramics (9); Materials Science Composites (7); Materials Science Multidisciplinary (7); Mechanics (7)	Materials (Ceramics/Composites) + Mechanics + Interdisciplinary Engineering/Applied Research
9	United States Air Force	Materials Science Composites (26); Materials Science Multidisciplinary (15); Materials Science Ceramics (14)	Materials (Composites/Ceramics) + Aerospace/Defense-oriented Engineering R&D (Interdisciplinary)
10	Beijing Institute of Technology	Materials Science Composites (13); Materials Science Multidisciplinary (8); Materials Science Ceramics (5); Mechanics (5)	Materials (Composites/Ceramics) + Mechanics + Interdisciplinary Engineering/Applied Research

## Data Availability

No new data were created or analyzed in this study. Data sharing is not applicable to this article.
